# Vaccination coverage and immunization timeliness among children aged 12-23 months in Senegal: a Kaplan-Meier and Cox regression analysis approach

**DOI:** 10.11604/pamj.supp.2017.27.3.11534

**Published:** 2017-06-21

**Authors:** Mouhamed Abdou Salam Mbengue, Aminata Mboup, Indou Deme Ly, Adama Faye, Fatou Bintou Niang Camara, Moussa Thiam, Birahim Pierre Ndiaye, Tandakha Ndiaye Dieye, Souleymane Mboup

**Affiliations:** 1 Institut de Recherche en Santé, de Surveillance Epidémiologique et de Formations (IRESSEF); 2 University of the Witwatersrand, Faculty of Health Sciences, School of Public Health, Johannesburg- South Africa; 3 Department of Preventive Medecine, University of Laval, Québec, QC, Canada; 4 Centre Hospitalier National d’Enfants Albert Royer, Faculty of Medecine, Cheikh Anta Diop University, Dakar, Senegal; 5 Department of Public Health, Faculty of Medecine, Cheikh Anta Diop University, Dakar, Senegal; 6 Agence National de la Statistique et de la Démographie, Dakar, Senegal; 7 Laboratory of Immunology, Cheikh Anta Diop University, Dakar, Senegal

**Keywords:** Vaccination, timeliness, immunization, children vaccine-preventable diseases, Senegal

## Abstract

**Introduction:**

Expanded programme on immunizations in resource-limited settings currently measure vaccination coverage defined as the proportion of children aged 12-23 months that have completed their vaccination. However, this indicator does not address the important question of when the scheduled vaccines were administered. We assessed the determinants of timely immunization to help the national EPI program manage vaccine-preventable diseases and impact positively on child survival in Senegal.

**Methods:**

Vaccination data were obtained from the Demographic and Health Survey (DHS) carried out across the 14 regions in the country. Children were aged between 12-23 months. The assessment of vaccination coverage was done with the health card and/or by the mother’s recall of the vaccination act. For each vaccine, an assessment of delay in age-appropriate vaccination was done following WHO recommendations. Additionally, Kaplan-Meier survival function was used to estimate the proportion vaccinated by age and cox-proportional hazards models were used to examine risk factors for delays.

**Results:**

A total of 2444 living children between 12–23 months of age were included in the analysis. The country vaccination was below the WHO recommended coverage level and, there was a gap in timeliness of children immunization. While BCG vaccine uptake was over 95%, coverage decreased with increasing number of Pentavalent vaccine doses (Penta 1: 95.6%, Penta 2: 93.5%: Penta 3: 89.2%). Median delay for BCG was 1.7 weeks. For polio at birth, the median delay was 5 days; all other vaccine doses had median delays of 2-4 weeks. For Penta 1 and Penta 3, 23.5% and 15.7% were given late respectively. A quarter of measles vaccines were not administered or were scheduled after the recommended age. Vaccinations that were not administered within the recommended age ranges were associated with mothers’ poor education level, multiple siblings, low socio-economic status and living in rural areas.

**Conclusion:**

A significant delay in receipt of infant vaccines is found in Senegal while vaccine coverage is suboptimal. The national expanded program on immunization should consider measuring age at immunization or using seroepidemiological data to better monitor its impact.

## Introduction

Immunization is amongst the most cost effective public health interventions for reducing global childhood morbidity and mortality [1]. In sub-Saharan African countries, where vaccine preventable diseases are the major contributors to high child mortality, vaccine timeliness is crucial to reverse this situation [2, 3]. World Health Organization (WHO) recommends that vaccines must be administrated during the first years of life within a specified schedule and time range [4]. The WHO recommended vaccination schedule aims to ensure to children suboptimal response, reduce individual vulnerability and prevent the recurrence outbreak of the diseases inside communities [4, 5]. Additionally, early protection is particularly important for haemophilus influenzae type b (hib) and pertussis which remains undiagnosed in most Low and Middle Income Countries (LMICs) with higher morbidity during infancy [6,7]. In sub-Saharan Africa, immunization data collected from administrative sources are inaccurate due to errors in the denominator (total target population), errors in recording vaccinations at health facilities, and errors in compiling the data on vaccinations to report to higher levels. To overcome this shortcoming, many countries obtain childhood immunization data by implementing a vaccination coverage cluster surveys such as Demographic and Health Survey (DHS) or a Multiple Indicator Survey (MICS) [8,9]. The most commonly used method is based on calculating vaccine coverage defined as the proportion of a given population that immunized in a given time period (e.g. by 12 or 23 months of age). However, despite current use, this method can only be calculated for a specified age group and does not cover delay to age-appropriate vaccination for a specific age group [10,11]. Additionally, studies have shown that high vaccination coverage do not necessarily correlates with vaccination timeliness and acquired immunity for children [11-14]. In Senegal, the infant mortality rate is high with 44 per 1000 live births and vaccine-preventable diseases are the leading cause of mortality amongst children under five years [15]. Senegal started its national EPI programme in 1976 with the principle to deliver vaccines through routine health services combined with regular mass distributions across the country. Currently, the EPI programme in Senegal recommends that a child receive BCG (Bacillus of Calmette-Guerin) vaccine against tuberculosis soon after birth. The oral polio vaccine (VPOo) and the monovalent pneumococcal conjugate vaccine (PCV) must also be administrated at birth and at 6, 10 and 14 weeks and at 9 months. Since 2005, the pentavalent vaccine has been introduced to replace DPT vaccine (diphtheria and tetanus toxoid with pertussis containing vaccine). The pentavalent vaccine contains a combination of 5 vaccines in one dose: diphtheria, tetanus toxoid, pertussis, hepatitis B, haemophilus influenza type b vaccine (Hib) and must be administered to children at the same time as the pneumococcal vaccine or the oral polio vaccine (6, 10, 14 weeks). In Senegal, like many developing countries where measles is highly endemic and frequently affect infants, routine measles vaccination is recommended at 9 months of age (Table 1). At 15 months, a second dose of measles is recommended to induce immunity in children who might have failed to mount a sufficient immune response to the first dose. Additionally, a dose of yellow fever is given at nine months [15]. In Senegal, despite relative high investment in vaccines and management of the national Expanded Programme on Immunization which resulted in increased immunization coverage in 2010 recurrent outbreaks of measles, diarrhea with rotavirus infection occurred among infants during the last decades at the national and regional level [16]. Therefore, some studies findings suggest that assessment of timely vaccination should be used to design and monitor vaccination programmes in low and middle income countries [17,18]. To our best knowledge, there is no study in Senegal exploring the exact time of immunization among children at the national level, and factors associated with immunization delay have been insufficiently investigated. Therefore, we conducted a secondary data analysis of the latest DHS to determine vaccination coverage and prevalence of timely vaccination for different vaccines administered within the national EPI programme. We also explored factors associated with time to vaccinations. We hypothesized that a better understanding of these determinants can potentially help the national EPI program manage vaccine-preventable diseases and positively impact child survival in Senegal.

**Table 1:** Recommended age for BCG, VPO, Penta, Rota, Pneumo, VPO, MR and AA vaccinations for children in Senegal and as per the WHO guidelines for childhood vaccinations

Recommended schedule with the National EPI programme	Vaccine	WHO recommended time range
At birth	BCG + VPO zero	Birth-4weeks
6 weeks	Penta 1 + Pneumo 1 + VPO1+ Rota1	4weeks - 2 months
10 weeks	Penta 2 + Pneumo 2 + VPO2+ Rota2	8 weeks – 4 months
14 weeks	Penta 3 + Pneumo 3 + VPO3	12weeks-6months
9 months	MR1 + VAA	38 weeks-12months
15 months	MR2	15-19 months

Penta: Pentavalent vaccine (DTC HiB, Hepatitis B); Pneumo: Pneumococcal conjugate vaccine. VAA: Vaccine antimalarial (yellow fever); RR: Measle rubella; BCG: Bacillus of Calmette and Guerin; VPO zero: Polio vaccine at birth; VPO1: Oral Polio Vaccine

## Methods

### Data source and sampling methodology

We used data from the latest DHS in Senegal where surveys data for the period of 2013-2014 is available upon request. The surveys were conducted in 2013 and 2014 across the 14 regions in Senegal. Data from each phase was aggregated to produce national data set with indicators statistically comparable across the regions. In general, the results from the DHS can be compared to previous standard DHS surveys. The objectives, organization, sample design of the Demographic and Health Survey are described elsewhere [19, 20]. Briefly, each phase of the DHS is a household survey with a two-stage stratified cluster sampling design. In the first stage, the primary sampling unit (PSUs) which is the census district was selected with probability proportional to PSU population size. In the second stage, households were selected and enumerated within each area segment. The sample was stratified by urban and rural areas, and the survey was carried out across the 14 regions. During each phase, information on child immunization was collected from the health card which recorded the child immunization history and the mother’s recall of vaccination. If the date of antigen administration was available recorded on the vaccination card, the information was directly collected. If the card indicated that an antigen was given but no date was recorded, the interviewer recorded the vaccination marked on the card. Mothers were asked to recall any vaccination that was given to the child but not listed in the card. For children without a health card, mother’s report of vaccination was accepted and recorded as valid information on childhood immunization status.

### Measurement of variables and descriptive statistics

We assessed vaccination coverage and timeliness of administration of each antigen according to the national immunization schedule implemented in Senegal. Up to date vaccination coverage in the country was assessed in children aged 12-23 months old as recommended by the World Health Organization. We defined vaccination coverage as the proportion of children aged between 12 and 23 months who received BCG, Polio 0, Polio 3, Penta 3, and Measles Containing Vaccine (MCV) vaccination [21]. Following WHO guideline and the definition in the EPI, a child was considered as fully vaccinated when he or she received 5 vaccines contact (BCG, 3 doses of polio vaccine expect the initial dose of oral polio vaccine given at birth), three doses of the pentavalent which contains 5 antigens against diphtheria, tetanus, pertussis, hepatitis B and haemophilus type b (Hib), and one dose of measles contained vaccine (Table 1). We generated the variable immunization status which was recoded as “1” if the child had received all 9 antigens and as 0 if not based on the health card or mother’s declaration. Time to vaccination for each vaccine was obtained from vaccination dates and date of birth recorded from the child health card. Following WHO guidelines, we defined immunization as correctly timed if the dose was administered within 4 weeks after the recommended age specified in the national immunization schedule (Table 1). In the situation where a vaccination was not recorded and the only the word “given” was mentioned, this data was considered as missing.

### Statistical analysis

Continuous variables were summarized by the mean and the standard deviation. Categorical variables were summarized by giving their frequencies. The analysis vaccination coverage was evaluated by using data recorded from health card and/or the mother's recall of the vaccination act. Participants included the analysis were children aged 12-23 months and having received BCG, Polio 0, Polio 3, Penta 3 and measles vaccines. We applied the non-parametric Kaplan-Meier method to estimate cumulative vaccination coverage at any given age. Assessment of delay in age-appropriate vaccination provides more information about timeliness of vaccination than up-to-date vaccination coverage and this method has been already described elsewhere [21-24]. In our analysis, time variable or survival time was defined as the age (in months) a child has survived until the vaccination at specified time. Thus, the failure or event of interest was a positive event defined as receipt of vaccine and the outcome variable was time in months until a child received a vaccine. Censoring occurred when for some children the observation period (0-23 months) ends while the child still does not received the specified vaccine. Another reason for censoring in survival analysis is that a person withdraws from the study because of death or some other reasons but this situation did not exist in our analysis as the question on uptake of vaccine during DHS was restricted to children alive at the time of the survey. For each vaccine, non-parametric Kaplan-Meier function of the time to event analysis was based on the following time ranges: BCG (Birth-8 weeks), Polio 0 (Birth-4weeks), three polio and the three pentavalent vaccines (4 weeks - 2months; 8 weeks - 4months; 12 weeks - 6 months) and measles vaccine (38 weeks-12 months). For each vaccine, we computed the cumulative event function at time t, denoted F(t) as the probability that the event has occurred by time t, or equivalently, the probability that the survival time is less than or equal to t. At any given age, the cumulative event function was estimated as the complement of the non-parametric Kaplan Meier estimate of the survivor function; F (t) =1-S (age). This method has been described elsewhere [11,23,25]. Finally, we applied a Cox proportional hazard model [25] which accounted for the complex survey design to determine the factors associated with vaccination delays for cinq (5) separates models: BCG, Oral Polio 0, Polio 3, Penta 3 and measles. We selected the adjustment-variables based on the WHO framework on epidemiology of the unimmunized [26]. The multivariate model was adjusted for the following variables: child’s gender, childhood place of residence, birth order of the child, child place of birth, mother’s education, marital status, and mother attending antenatal care, wealth index, and regions of residence.

### Ethical clearance

Ethical approval was granted by the Ethics Committee of the National Statistical Office of Senegal. The 2012-2013-2014 continuous DHS data is available to the general public by request in different formats from the Measure DHS website (www.measuredhs.com). We submitted a request to the Measure DHS by describing the purpose and objectives of the study and received permission to download the children’s data set in STATA format.

## Results

### Characteristics of the study population

A total of 2,444 living children between 12-23 months of age were included in the analysis for the vaccination coverage. The mean age of the children was 17 months. 52.8% were female and more than (60.8%) half of sampled children were living in rural area. The percentage of mother with no education was high (68%) and 93.1% of them were married. Almost all the mothers attended antenatal care (96. 54%).The proportion of children with available vaccination cards was 69.74% and 23.77% of children were in the poorest quintile of the wealth index (Table 2).

**Table 2:** Demographic characteristics of the study population

Child's characteristics	N	Proportion
**Gender**		
Male	1,170.93	47.92
Female	1,272.41	52.08
**Childhood place of residence**		
Urban	957.92	39.21
Rural	1,485.44	60.79
**Birth order of child**		
0-3	1,517.72	79.97
4-6	304.81	16.06
>=7	75.41	3.97
**Child place of birth**		
At home	616.11	25.22
at health care facility	1,827.23	74.78
**Mother's education**		
No education	1,658.98	67.90
Up to primary	520.13	21.29
Secondary and higher	264.22	10.81
**Marital status**		
married	2,274.85	93.10
others	168.48	6.90
**Vaccination card available**		
yes	1,703.91	69.74
No	739.42	30.26
**Mother attended antenatal care**		
yes	2,197.48	96.54
No	78.72	3.46
**Wealth index**		
Poorest	580.87	23.77
Poorer	532.57	21.80
Middle	494.73	20.25
Richer	421.55	17.25
Richest	413.60	16.93
**Region**		
Dakar	408.15	16.70
Ziguinchor	76.72	3.14
Diourbel	261.87	10.72
Saint-Louis	216.39	8.86
Tambacounda	144.11	5.90
Kaolack	238.54	9.76
Thies	364.49	14.92
Louga	154.40	6.32
Fatick	110.41	4.52
Kolda	122.73	5.02
Matam	103.72	4.25
Kaffrine	137.02	5.61
Kedougou	32.64	1.34
Sedhiou	72.09	2.95

### Vaccination coverage

Overall, the prevalence of full immunized children was 72.2% (95%CI: 69.2-74.8%). In Dakar, the capital city of Senegal, this proportion was 86.4% (95%CI: 75%-92%). Tambacounda and Kolda had the lowest immunization coverage with respectively 54% (95%CI: 50.6-73) and 56% (95%CI: 54.4%-75%). Except Dakar, the highest immunization coverage rate was found in Fatick with 82.49% (Table 3). The vaccination coverage for BCG and Penta3 was 95.8% and 89.2% respectively. A high coverage of BCG was achieved in all geographical regions. Coverage of the BCG vaccination ranged from 100% in Dakar to 75.12% in Matam. For the polio vaccine at birth and Polio 3 vaccine, the vaccination coverage was 70% and 83.7% respectively. Coverage of last dose of Polio (Polio3) ranged from 98% in Ziguinchor to 77.59 % in Tambacounda. Coverage for two doses of Penta 1 and Penta 2 ranged from 89.77% to 93.44%. The coverage for the DPT3 dose was lower in Kolda (65.04%) compared to 97.8% in the region of Ziguinchor. The vaccination coverage with MCV was less than 80% among children aged 12-23 months. Regarding the distribution per region, measles immunization coverage ranged from 89.57% in Saint-Louis to 64.6% in Tambacounda (Table 3).

**Table 3:** Immunization coverage among children aged 12-23

Vaccine weighted	respondent(n)	Immunization n(%)
BCG	2,443	2,340.80 (95.8%)
Polio 0	2,443	1,728.7 (70.7%)
Polio 1	2,443	2,342.72 (95.8%)
Polio 2	2,443	2,271.22 (92.9%)
Polio 3	2,443	2,046.9 (83.7%)
Pentavalent 1	2,443	2,337.8 (95.6%)
Pentavalent 2	2,443	2,284.03 (93.5%)
Pentavalent 3	2,443	2,179.48 (89.2%)
Measle	2,443	1,935.3 (79.2%)
All antigens	2,443	1,762.8 (72.2%)

BCG = Bacillus Calmette-Guerin; Penta = diphtheria, pertussis, tetanus, haemophilus influenza b and hepatitis B vaccine

OPV = oral polio vaccine; n=weighted total number of children

1 Polio 0 is the polio vaccination given at birth

2 BCG, measles, and three doses of Penta and Polio vaccine (excluding oral polio vaccine at birth)

### Time in correctly timed vaccination

Approximatively, 30% of infants aged 12-23 months did not have a vaccination card at the time of the survey. Therefore, to study timeliness of receiving each antigen, we restricted our analysis to 1,704 children aged 12-23 months whose mothers showed a vaccination card during the interview. For BCG, the mean age of immunization was 1 months and 88.25 % of children aged 12-23 months received the BCG vaccine within the recommended time schedule (between 0-4 weeks) (Table 4). Median delay for BCG was 1.7 weeks. For polio vaccine at birth, the median delay was 5 days; all other vaccine doses had median delays of 2-4 weeks. For Penta 1 and Penta 2 the proportion of children with timely vaccination was respectively 73, 84% and 75, 64%. For polio 3 vaccine, the median age at vaccination was 5.22 months and 15.71% of the vaccinations were given late. Overall, the median age at vaccination for MCV was 9.73 months and the proportion of children with correctly timed vaccination was 72.3% (Table 4).

**Table 4:** Number vaccinated, missing dates and timeliness of each vaccine among children with child health cards

Vaccine	Not received	Received, no date	Timeliness of each vaccine	
	n (%)	n (%)	Timely	Late
**BCG**	24.81 (1.46 %)	50 (2.93%)	1,503.6 (88.25%)	125.4 (7.36%)
**Polio 0**	429.9 (25.23%)	194.16 (11.40%)	958.78 (56.27%)	121.03 (7.10%)
**Polio 1**	15.27 (0.90%)	26.073 (1.53%)	1,258.8 (73.88%)	403.7 (23.69%)
**Polio 2**	56.40 (3.31%)	40.78 (2.39%)	1,280.8 (75.17%)	325.8 (19.12%)
**Polio 3**	126.32 (7.41%)	58.58 (3.44%)	1,251.2(73.44%)	267.7 (15.71%)
**Pentavalent 1**	18.21 (1.07%)	26.29 (1.54%)	1,258.2 (73.84%)	401.2 (23.55%)
**Pentavalent 2**	53.75 (3.15%)	40.47 (2.38%)	1,288.8 (75.64%)	320.8 (18.83%)
**Pentavalent 3**	119.69 (7.02%)	53.68 (3.15%)	1,255.5 (73.69%)	274.98 (16.14%)
**Measles**	293.31 (17.21%)	84.28 (4.95%)	1,231.7(72.29%)	94.62 (5.55%)

### Cumulative vaccination coverage based on Kaplan-Meier Method

**Figure 1:**
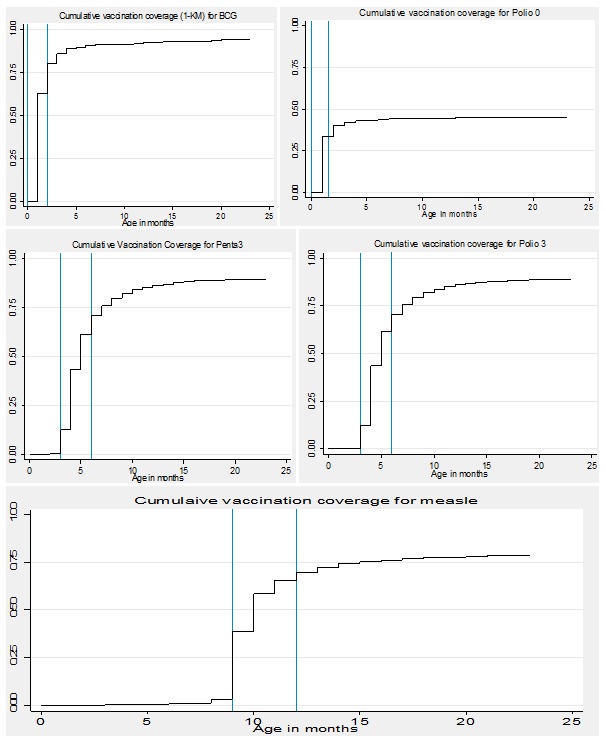
Cumulative incidence coverage (1-KM) for each antigen

Vaccination coverage estimates with the Kaplan-Meier method are shown in Figure 1. The time course of completion of Penta 3 and Polio 3 vaccine are described in Figure 1. For Penta 3 and Polio 3 vaccine completion, recommended by month 3 is achieved in approximately 10% of the children. It takes about 5 months for 50% of children to complete the pentavalent and the polio vaccine. For both vaccines, there is a rise in vaccination coverage from 6 to 23 months indicating noticeable delays when compared to the WHO guidelines. For measles coverage, where the WHO’s specified time schedule ranges from 9 to 12 months, 50% of children received the MCV around11 months, and less than 70% coverage rate was achieved during the first year of life. Similarly, the cumulative incidence curve rose from 12 to 23 months indicating considerable delay in measles vaccination (Figure 1).

### Factors associated with time to start of vaccination

Female children were more likely to have correctly timed vaccinations with MCV, Penta 3 and Polio 3. Birth order of the child was associated with vaccination with Polio 3 and Penta 3. Children of mothers with a primary education level were 22% more likely to receive the Penta 3 vaccine higher at a given age compared to children born to women with no education at that age. Similarly, women with secondary education or higher were 20% more likely to receive Penta 3 vaccine as compared to women without education level (Table 5). In Senegal, living in urban areas was associated with delays with both Penta 3 and MCV vaccinations. Regional differences in timely vaccinations were found in many regions. In Diourbel, Tambacounda, Kolda and Sedhiou Polio and MCV vaccinations were more likely to be delayed than in the capital city, Dakar. Fatick and Ziguinchor, the association was reversed i.e. Penta 3 vaccination was more likely to be delayed in Dakar, the capital city, compared to other regions (Table 6).

**Table 5:** Predictors of untimely vaccinations with the Cox regression

Demographics	BCG	Polio 0	Polio 3	Penta 3	MCV
	HR (95% CI)	HR (95% CI)	HR (95% CI)	HR (95% CI)	HR (95% CI)
**Gender**					
Male	1	1	1	1	1
Female	0.98 (0.88-1.10)	1.05 (0.90 -1.2)	1.08 (0 .98-1.1)	1.12 (1.10- 1.2) *	1.12 (1.00-1.27) *
**Place of residence**					
Urban	1	1	1	1	1
Rural	0.91 (0.76-1.07)	0.85 (0.70-1.04)	0.89 (0.78-1.02)	0.89 (0.8-1.02)	1.00(0.84-1.19)
**Birth order of child**					
0-3	1	1	1	1	1
= 4-6	0 .99 (0.85-1.15)	1.04 (0.90-1.30)	1.21 (1.07-1.37) *	1.2(1.10-1.40) *	1.11 (0.94-1.30)
>=7	0.96 (0.71-1.31)	0.74 (0.50-1.12)	1.27 (1.01-1.59) *	1.3(1.02-1.61) *	1.03 (0.74 -1.41)
**Place of delivery**					
At home	1	1	1	1	1
at health care facility	1.03 (0.90-1.18)	1.02 (0.88-1.18)	1.03 (0.90 -1.16)	1.01 (0.90 -1.15)	0.97 (0.80-1.13)
**Mother's education**					
No education	1	1	1	1	1
Up to primary	0 .95 (0.80 -1.10)	0.97 (0.80 -1.20)	1.22 (1.08-1.40) *	1.18 (1.05 -1.30)*	1.11 (0.95-1.30)
Secondary and higher	1.08 (0.87 -1.36)	1.2 (0.90 -1.50)	1.22 (1.01-1.48) *	1.16 (0.90 -1.40)*	0.86 (0.65-1.14)
**Marital status**					
Married	0.99 (0.70 -1.30)	1.07 (0.70 -1.61)	0.97 (0.70 -1.26)	0.96 (0.75-1.25)	1.24 (0.80-1.70)
Others	1	1	1	1	1

**Table 6:** Predictors of untimely vaccinations with the Cox regression (Continued)

Demographics	BCG	Polio 0	Polio 3	Penta 3	MCV
	HR (95% CI)	HR (95% CI)	HR (95% CI)	HR (95% CI)	HR (95% CI)
**Attended four ANC**					
Yes	1.12 (0.80-1.50)	1.10 (0.70 -1.80)	1.08 (0.80 -1.50)	1.12 (0.80 -1.50)	1.00 (0.67-1.47)
No	1	1	1	1	1
**Wealth index**					
Poorest	1	1	1	1	1
Poorer	0.98 (0.83 -1.14)	0.90 (0.80 -1.20)	1.14 (0.98 -1.32)	1.16 (1.10-1.35) *	0.99 (0.82-1.19)
Middle	1.05 (0.80 -1.28)	1.12(0.80-1.44)	1.35 (1.15 -1.59) *	1.38 (1.20-1.60) *	1.12 (0.90 -1.10)
Richer	1.07 (0.80 -1.34)	1.20 (0.90-1.60)	1.25 (1.03 -1.51) *	1.26 (1.10-1.53) *	0.70 (0.50 - 0.91) *
Richest	1.50 (1.20-1.95) *	1.20 (0.80-1.60)	1.49 (1.21-1.84) *	1.49 (1.20-1.84) *	0.96 (0.73 -1.26)
**Region**					
Dakar	1	1	1	1	1
Ziguinchor	1.57 (1.10-2.20)	0.61 (0.40 - 0.90)	1.13 (0 .84 - 1.52)	1.20 (0.90 - 1.61)	1.00 (0.67 - 1.48)
Diourbel	1.21 (0.93 -1.58)	0.81 (0.60 -1.10)	0.84 (0.68 - 1.03)	0.80 (0.70 - 1.08)	0.80 (0.59 - 1.03)
Saint-louis	1.63 (1.20 -2.10)	0.28 (0.20 - 0.40)	1.05 (0.85 - 1.30)	1.12 (0.90 - 1.38)	1.01 (0.70 - 1.32)
Tambacounda	0.99 (0.70 -1.38)	0 .40 (0.20 - 0.70)	0.63 (0.47- 0.84)	0.64 (0.48 - 0.80)	0.55 (0.40 - 0.80) *
Kaolack	1.17 (0.90 - 1.50)	0.50 (0.40 - 0.69)	0.82 (0.70 - 1.01)	0.84 (0.68 - 1.04)	0.72 (0.50 - 0.96) *
Thies	1.34 (1.00 - 1.72)	0.80 (0.60 -1.10)	1.13 (0.90 -1.35)	1.10 (0.94 - 1.35)	0.75 (0.60 - 0.97) *
Louga	1.43 (1.00 -1.95)	0.40 (0.30 - 0.65)	0.88 (0.68 -1.13)	0.89 (0.70 - 1.14)	0.77 (0.55 - 1.07)
Fatick	1.51 (1.10 -2.07) *	0.90 (0.70 - 1.40)	1.09 (0.84 - 1.41)	0.15 (0.80 - 1.40)	0.78 (0.55 - 1.10)
Kolda	1.35 (0.90 -1.84)	0 .43(0.30 - 0.60)	0.90 (0.68 - 1.18)	0.89 (0.70 - 1.18)	0.72 (0.50 - 1.03)
Matam	1.21 (0.80 - 1.67)	0.41 (0.30 - 0.60)	0.94 (0.71 - 1.23	0.94 (0.73 -1.24)	0.79 (0.50 - 1.13)
Kaffrine	1.31 (0.90 -1.80)	0.37 (0.20 -0.60)	0.88 (0.67 - 1.16)	0.96 (0.73 -1.26)	0.76 (0.50 - 1.09)
Kedougou	1.01 (0.50 -1.70)	0.24 (0.10 - 0.70)	1.11 (0.70 - 1.84)	1.10 (0.66 - 1.83)	0.78 (0.40 - 1.56)
Sedhiou	1.52 (1.00 - 2.24)	0.50 (0.30 - 0.80)	1.28 (0.94 - 1.77)	1.30 (0.94 - 1.79)	0.85 (0.55 - 1.30)

## Discussion

Despite relative high vaccination coverage based on health card or mother’s recall of vaccination, there was a gap in the timeliness of children immunization and almost 25% of measles contained vaccine were not received or scheduled after the recommended age. For Penta 1 and Penta 3, 23.6% and 15.7% were given late respectively. Untimely vaccination was associated with male gender, birth order, and education level and (not significant). Our findings confirm previous studies in sub-Saharan and low middle income countries that reported high rate of delay in age-appropriate vaccination despite high vaccination coverage based on vaccination card or mother’s recall of immunization [27-29]. WHO considers high BCG uptake as real marker of good access to health care system for pregnant women (30). In our study, the proportion of infants not receiving BCG vaccine was < 2% and only 7.4% received the BCG vaccine after one month. This may suggest that globally during the first months of life, health centers are well performing vaccine delivery. In our study, there was a steady increase of delay in age-appropriate vaccination for BCG and Penta 3. Our findings confirm previous studies [30] that have also reported an increase in the time to immunization for vaccines given after birth compared to vaccine received later when children get older. This situation may be explained by the different factors related to vaccine logistics for certain regions. Another reason might be that some mothers or guardians may not choose to come back after an adverse event following the first immunization. In a meta- analysis review Munoz, et al. (2015) [30] showed that in low and middle countries concerns about vaccinations are widespread and further worsen the challenge related to programmatic and health system barriers to vaccination [30]. In many sub-Saharan countries, the use of vaccination services might have been slowed by the replacement of traditional vaccines with expensive combinations which may curve the vaccine wastage and the timeliness [16]. Another cause of delay in age appropriate vaccination may be the increasing complexity of the immunization schedule. Like other low and middle-income countries, in Senegal, routine immunization schedule has gone beyond the traditional childhood vaccines since its inception in 1976. At the beginning the national Expanded Programme on Immunization recommended delivering only six antigens (BCG, which traditionally includes diphtheria, tetanus, pertussis, measles polio and tuberculosis). Since 2012, Senegal has progressively introduced into its EPI program, hepatitis b, Hib, pentavalent vaccine against Hib and rotavirus vaccine. For measles, the second dose of the vaccine is offered through the national immunization programme to children beyond their first birthdays. Consequently, delayed immunization may be caused by the fact that some health care providers are uncertain on how to treat children who have missed out previously scheduled vaccine or don’t know how to plan a catchup schedule. According to the external review of the national immunization programme in 2010, only 58% of health districts have delivered formal EPI training programme during the last 3 years. Given the complexity and the evolving change of the EPI recommended-vaccines there is a necessity for training, supervision and a continuous education programme among health professionals. Measles disease is highly transmissible with high morbidity and mortality. The WHO- UNICEF global immunization vision and strategy 2006-2015 aimed at controlling morbidity and mortality from vaccine-preventable diseases recommends to country to attain at least 90% measles immunization coverage [5]. In our study, less than 70% coverage rate was achieved during the first year of life and the cumulative incidence curve showed considerable delay in measles vaccination after 12 months. Our results confirm previous studies in sub-Saharan Africa which shows considerable delays in administration of measles administration after 12 months [11,24]. In 2009, a massive outbreak occurred in Senegal with continuous virus transmission from late 2009 to early 2010 [16, 31]. During this outbreak, it was confirmed that 11,3% of affected children did receive a measles contained vaccine but was not correctly immunized. Previous studies in sub-Saharan Africa have shown that relatively low vaccination coverage rates is the main cause of measles circulation because children are not immunized or under immunized [17]. Our results show that children from the highest wealth quintiles were more likely to be vaccinated on time compared to children from the lowest wealth quintile. A study of 31 low and middle-income countries also found that children in poorer families and families with more than one child were at increased risk for vaccination delay [17]. In a study in Ethiopia, Lakew et al [32] showed that children born to mothers of higher wealth index were 40% more likely to be fully vaccinated compared to children from women of poor wealth index group. Babirye et al [23] found in Uganda similar substantial differences in timeliness across educational and socio-economic classes. Our study is not without limitations. Firstly, because of potential shame and social stigma, mothers of children who don’t have the vaccination recorded on the health card may be more tempted to report a vaccination for their children introducing a potential subject bias. Consequently, the level of immunization coverage may be lower than the prevalence reported in this study Secondly, we used only information that was recorded on vaccination card to obtain accurate vaccination and delay in age-appropriate vaccination. Although this was the most accurate method to obtain vaccination dates, parental cards are also subject to incompleteness and some dates may have not been written in the vaccination cards, resulting in an overestimate of the prevalence of delay for each antigen within the national EPI programme. Vaccination coverage was only estimated among children born two years prior the survey and children who died or out –migrated were not recorded during the household survey, this situation might have been a potential bias in estimates of the cumulative proportion. We used 1 minus the Kaplan-Meier function to estimate the proportion vaccinated by age consistent with previous studies. This method is an easy and useful way of visualizing the vaccination uptake over time (or age) and provides estimates of the proportion vaccinated at a given age, which may be useful in assessing the performance of vaccination programs in reaching their targets. However, this method will consistently give higher results than conventional methods due to censoring, which reduces the population at risk at the time point when censoring occurs. Also, as the number of persons under observation decreases with time, the rightward part of the curve becomes unstable.

## Conclusion

We found significant delays in receipt of recommended infant vaccinations in this nationally representative sample and socio-demographic and regional inequalities in vaccination timing. Our results imply that for the national EPI programme to have a real impact vaccine preventable disease, timeliness of routine childhood immunization should be emphasized. In addition, innovative strategies to improve timeliness of childhood immunization combined with the reinforcement of the current laboratory-based surveillance system must be established in Senegal.

### What is known about this topic

- Current studies on childhood vaccines focus on the proportion of children having completed their vaccination in the group aged 12-23 months in Senegal and other sub-Saharan countries;
- Although it appears that the magnitude of immunoglobulins antibody responses that is given by a EPI vaccine is directly related to age at immunization, there is limited data on timeliness of childhood immunization in Senegal and other sub-Saharan countries;
- Measuring vaccine coverage only does not address the important question on when the scheduled vaccines were administered.

### What this study adds

- Our study uses national representative household survey to provide evidence on immunization delay among children aged 12-23 months in Senegal;
- Our findings show that despite high reported vaccination coverage based on health card or mother’s recall of vaccination, there is a gap in the timeliness of children immunization;
- Our finding emphasizes that in Senegal and other sub-Saharan countries, national EPI programme, should focus on innovative strategies to improve immunization timeliness among children aged 12-23 months.

## Competing interests

The authors declare no competing interest.
